# Phosphorylation of TGB1 by protein kinase CK2 promotes barley stripe mosaic virus movement in monocots and dicots

**DOI:** 10.1093/jxb/erv237

**Published:** 2015-05-21

**Authors:** Yue Hu, Zhenggang Li, Cheng Yuan, Xuejiao Jin, Lijie Yan, Xiaofei Zhao, Yongliang Zhang, Andrew O. Jackson, Xianbing Wang, Chenggui Han, Jialin Yu, Dawei Li

**Affiliations:** ^1^State Key laboratory of Agro-Biotechnology and Ministry of Agriculture Key Laboratory of Soil Microbiology, College of Biological Sciences, China Agricultural University, Beijing 100193, PR China; ^2^Department of Plant and Microbial Biology, University of California-Berkeley, Berkeley, CA 94720, USA

**Keywords:** Barley stripe mosaic virus, triple gene block 1 (TGB1) protein, phosphorylation, protein kinase CK2, promotion, viral movement.

## Abstract

The barley stripe mosaic virus (BSMV) triple gene block 1 (TGB1) protein is required for virus cell-to-cell movement. However, little information is available about how these activities are regulated by post-translational modifications. In this study, we showed that the BSMV Xinjiang strain TGB1 (_XJ_TGB1) is phosphorylated *in vivo* and *in vitro* by protein kinase CK2 from barley and *Nicotiana benthamiana.* Liquid chromatography tandem mass spectrometry analysis and *in vitro* phosphorylation assays demonstrated that Thr-401 is the major phosphorylation site of the _XJ_TGB1 protein, and suggested that a Thr-395 kinase docking site supports Thr-401 phosphorylation. Substitution of Thr-395 with alanine (T395A) only moderately impaired virus cell-to-cell movement and systemic infection. In contrast, the Thr-401 alanine (T401A) virus mutant was unable to systemically infect *N. benthamiana* but had only minor effects in monocot hosts. Substitution of Thr-395 or Thr-401 with aspartic acid interfered with monocot and dicot cell-to-cell movement and the plants failed to develop systemic infections. However, virus derivatives with single glutamic acid substitutions at Thr-395 and Thr-401 developed nearly normal systemic infections in the monocot hosts but were unable to infect *N. benthamiana* systemically, and none of the double mutants was able to infect dicot and monocot hosts. The mutant _XJ_TGB1_T395A/T401A_ weakened *in vitro* interactions between _XJ_TGB1 and _XJ_TGB3 proteins but had little effect on _XJ_TGB1 RNA-binding ability. Taken together, our results support a critical role of CK2 phosphorylation in the movement of BSMV in monocots and dicots, and provide new insights into the roles of phosphorylation in TGB protein functions.

## Highlight

CK2 phosphorylation of the TGB1 protein has a critical role in promoting barley stripe mosaic virus movement in monocots and dicots by affecting the interactions between TGB1 and TGB3 proteins.

## Introduction

*Barley stripe mosaic virus* is the type species of the genus *Hordeivirus*. Barley stripe mosaic virus (BSMV) infects barley, wheat, and oats under natural conditions, and numerous other monocots and dicots by artificial inoculation (Bragg *et al.*, 2008; Jackson *et al.*, 2009). BSMV has recently been isolated from 750-year-old barley grains found near the Nile River (Smith *et al.*, 2014), and consists of a large number of strains that were early subjects of phenotypic and host-range studies (McKinney and Greeley, 1965). BSMV has a positive-sense tripartite RNA genome (RNAα, -β, and -γ) that encodes seven major proteins (Bragg *et al.*, 2008; Jackson *et al.*, 2009). RNAα directs synthesis of the αa protein, which functions as the methyltransferase/helicase subunit of the replicase [RNA-dependent RNA polymerase (RdRp)]. RNAβ encodes βa [coat protein (CP)], and an overlapping triple gene block (TGB) sequence encoding three major movement proteins (TGB1, TGB2, and TGB3) that are expressed from two subgenomic RNAs. RNAγ serves as the mRNA for the γa RdRp polymerase subunit, and the γb protein, which functions as a suppressor of RNA silencing and a modulator of host defences (Jackson *et al.*, 2009).

The BSMV TGB1 protein is a multifunctional 58kDa protein with RNA-binding, RNA helicase, and ATPase activities (Donald *et al.*, 1997; Verchot-Lubicz *et al.*, 2010). The C-terminal region contains seven conserved helicase motifs, and mutations within one or more of these motifs have been shown to be involved in enzymatic and movement functions and RNA-binding activities (Lawrence and Jackson, 2001; Lim *et al.*, 2008). TGB2 and TGB3 are trans-membrane proteins that are integrated in the endoplasmic reticulum bilayer (Morozov and Solovyev, 2003). Cell-to-cell movement of BSMV does not require the CP, and this property has permitted isolation of nucleoprotein complexes composed of the TGB1 protein and viral genomic and subgenomic RNAs (Lim *et al.*, 2008). The TGB1 protein interacts directly with the TGB3 protein and indirectly with the TGB2 protein to form heterologous complexes required for co-localization of the TGB proteins at the plasmodesmata (PD) and BSMV cell-to-cell movement (Lawrence and Jackson, 2001; Lim *et al.*, 2008; Jackson *et al.*, 2009; Lim *et al.*, 2009). Our recent results have shown that TGB1 proteins function in eliciting resistance to BSMV strains that are unable to infect *Brachypodium distachyon* inbred lines containing the *Bsr1* resistance gene. In the case of the North Dakota 18 (ND18) strain TGB1 protein, amino acid residues at positions 390 and 392 are critical for TGB1 protein genetic interactions with *Bsr1* and for inducing resistance responses (Cui *et al.*, 2012; Lee *et al.*, 2012).

Although the studies mentioned above provide valuable insights into BSMV cell-to-cell movement processes, little information is available about post-translational biochemical events, such as phosphorylation, that may function in regulating intercellular macromolecular trafficking. Movement protein (MP) phosphorylation was first demonstrated to be important for virus cell-to-cell movement during investigations with the tobacco mosaic virus (TMV) 30kDa MP, and this study provided important approaches for subsequent MP analyses (Citovsky *et al.*, 1993). Several proteins may function in TMV movement, because a cell-wall-associated kinase (Citovsky *et al.*, 1993), a PD-associated protein kinase (PAPK1) (Lee *et al.*, 2005), an endoplasmic reticulum-associated kinase (Karger *et al.*, 2003), and protein kinase CK2 (formerly known as casein kinase II; Ivanov *et al.*, 2003) have been shown to be involved in *in vitro* and *in vivo* phosphorylation of the 30kDa protein (Citovsky *et al.*, 1993). Moreover, mimicking MP phosphorylation by negatively charged amino acids inhibited MP transport through PD and delayed TMV and potyvirus spread in *Nicotiana tabacum* (Waigmann *et al.*, 2000; Karger *et al.*, 2003). More recently, phosphorylation of the MPs of other viruses, such as tomato mosaic virus (Kawakami *et al.*, 2003; Matsushita *et al.*, 2003), potato leafroll virus (Link *et al.*, 2011), Abutilon mosaic virus (Kleinow *et al.*, 2009), brome mosaic virus (Akamatsu *et al.*, 2007), apple chlorotic leaf spot virus (Sato *et al.*, 1995), and cucumber mosaic virus (Matsushita *et al.*, 2002), has been shown to either enhance or inhibit virus movement during infection.

Although little information is available about the roles of phosphorylation in the movement processes of TGB MPs, potato virus X TGB1 protein is efficiently phosphorylated by *N. tabacum* protein kinase CK2 (Módena *et al.*, 2008). Furthermore, tyrosine phosphorylation regulates the functions of potato mop-top virus TGB3 protein because substitution of tyrosine residues in two phosphorylation domains enhances interactions between the TGB3 and TGB2 proteins and inhibits virus cell-to-cell movement (Samuilova *et al.*, 2013). In addition, the N-terminal half of the TGB1 protein of Poa semilatent virus (PSLV), a hordeivirus closely related to BSMV, has been reported to be phosphorylated *in vitro* by casein kinase 1 (CK1), protein kinase A (PKA), and protein kinase C (PKC)-like kinases present in *N. benthamiana* cell-wall fractions. In the case of PSLV, phosphorylation of an internal domain decreases RNA-binding activity and homologous protein–protein interactions, but experiments to determine whether these activities affect movement have not been conducted (Makarov *et al.*, 2012). Here, we present the first evidence that BSMV TGB1 protein is phosphorylated *in vitro* and *in vivo* by the host protein kinase CK2. Our biochemical and molecular approaches demonstrated that Thr-401 in the TGB1 C-terminal region is a major phosphorylation site of the TGB1 protein, and that the Thr-395 residue serves as a CK2 docking domain. Mutational analyses of these residues indicate that a phosphorylation-dependent mechanism is involved in BSMV local and systemic infections in monocots and dicots.

## Materials and methods

### Plant growth conditions

*N. benthamiana* plants were grown in a climate chamber at 23 to 25 °C with a 14/10h light (~75 mmol m^–2^ s^–1^)/dark photoperiod as described previously (Yuan *et al.*, 2011). Barley (Yangpi 8), wheat (Yangmai 11), and *B. distachyon* Bd21 plants were grown in a greenhouse until the two-leaf stage, and then inoculated and transferred to a climate chamber at the same temperature and light regimen as above until evaluated at 5 to 12 d post-inoculation (dpi).

### Construction of infectious clones of BSMV Xinjiang (_XJ_BSMV) strain

Genomic (g) RNAs of _XJ_BSMV strain were extracted from purified virus with Trizol (Life Technologies) and used to prime reverse transcription of the gRNAs with primer BS32 as described previously (Yuan *et al.*, 2011; Lee *et al.*, 2012). BSMV α, β, and γ cDNAs were amplified with the primer pairs XJ-1/BS32, XJ-2/BS32, and XJ-3/BS32, respectively (Supplementary Table S1, available at *JXB* online), and inserted into the pMD20-T vector (Takara) to generate pT7-α_XJ_, pT7-β_XJ_, and pT7-γ_XJ_. Site-specific mutagenesis was carried out with a QuikChange Site-Directed Mutagenesis kit (Agilent Technologies) to make alanine (A), aspartic acid (D), and glutamic acid (E) substitutions for _XJ_TGB1 protein residues 395 and 401 in pT7-β_XJ_ with the corresponding primer pairs (Supplementary Table S2, available at *JXB* online). These clones and all those described below were verified by DNA sequencing (Tsingke Biotech, Beijing).

To engineer _XJ_BSMV derivatives for agroinfiltration, full-length cDNAs were amplified from pT7-α_XJ_, pT7-β_XJ_ (or the site-specific pT7-β_XJ_ TGB1 mutants), and pT7-γ_XJ_ clones with the primer pairs CH-10/BS-26, CH-11/BS-26, and CH-12/BS-26, respectively (Supplementary Table S1). The cDNAs then were inserted between the *Stu*I and *Bam*HI sites of pCass4-Rz (Annamalai and Rao, 2005), and the resulting clones and site-specific mutants with the 395 and 401 residue substitutions were designated pCa-α_XJ_, pCa-β_XJ_, pCa-γ_XJ_, and pCa-β_XJ_TGB1 mutants.

### Mechanical inoculation of *in vitro* transcripts and agroinfiltration of BSMV derivatives

The pT7-α_XJ_, pT7-β_XJ_, and pT7-γ_XJ_ plasmids were linearized with *Spe*I or *Bam*HI and used as templates for T7 RNA polymerase (Promega) *in vitro* transcription of capped infectious RNAs (Petty *et al.*, 1989). The α, β, and γ *in vitro* transcripts were mixed in equal amounts with FES inoculation buffer (0.1M glycine, 0.06M potassium phosphate, 1% sodium pyrophosphate decahydrate, 1% bentonite, 1% celite, pH 8.5) and used immediately for inoculation of 7- to 10-d-old barley, wheat, and *B. distachyon* Bd21. Plasmids pCa-α_XJ_, pCa-β_XJ_, and pCa-γ_XJ_ were maintained in *Agrobacterium tumefaciens* strain EHA105 and infiltrated into the lower side of *N. benthamiana* leaves as described previously (Yuan *et al.*, 2011).

### Immunoprecipitation

Immunoprecipitation assays were carried out with minor modifications of a published protocol (Rubio *et al.*, 2005). *N. benthamiana* leaf sections were harvested at 5 d after agroinfiltration and proteins were extracted in 2 vols (w/v) of GTEN buffer [10% (v/v) glycerol, 25mM Tris/HCl (pH 7.5), 1mM EDTA, 150mM NaCl, 10mM dithiothreitol, 2% (w/v) polyvinylpolypyrrolidone, 1% Protease Inhibitor Cocktail (Roche)]. Extracted complexes were stirred for 30min and centrifuged at 12 000*g* for 30min at 4 °C. The supernatants were first incubated with TGB1 protein antibody at 4 °C for 6h, and then mixed with protein G–agarose (Millipore) beads to enrich the _XJ_TGB1 protein. The immunoprecipitation products were washed five times with immunoprecipitation buffer [GTEN buffer with 0.15% (v/v) NP-40, 0.5mM dithiothreitol] and evaluated by Western blotting with anti-TGB1 and anti-phosphothreonine antibody (α-pT; Millipore) at 1:500 dilutions.

### Construction of protein expression vectors for *in vitro* phosphorylation, glutathione S-transferase (GST), and His-tagged pull-down assays

To engineer _XJ_TGB1 C-terminal His-tagged (_XJ_TGB1-6His) fusion proteins, the _XJ_TGB1 open reading frame (ORF) was amplified from the plasmid pT7-β_XJ_ with the primer pair TGB1-NdeIF/TGB1-XhoIR (Supplementary Table S1), and integrated into the *Nde*I and *Xho*I sites of the pET-30a(+) expression vector (Novagen) to generate pET-_XJ_TGB1. The TGB1 mutant derivatives of the Thr-395 and Thr-401 residues were engineered using a QuikChange Site-Directed Mutagenesis kit with corresponding primer pairs for the TGB1 ORFs (Supplementary Table S2).

The α-catalytic subunit of CK2 from *N. benthamiana* (NbCK2α, GenBank accession no. KJ748371) (Bombarely *et al.*, 2012) and barley (HvCK2α, GenBank accession no. AB252049) (Kato *et al.*, 2008) was isolated from host RNA by reverse transcription (RT)-PCR amplification with the primer pairs NbCK2-NdeIF/NbCK2-XhoIR and HvCK2α-NdeIF/HvCK2α-SalIR, respectively (Supplementary Table S1). Primers for amplifying NbCK2α were based on the flanking sequence of the *N. tabacum* CK2α ORF (GenBank accession no. AF374474) (Salinas *et al.*, 2001). The PCR products of the NbCK2α and HvCK2α genes were inserted into the *Nde*I/*Xho*I, and *Nde*I/*Sal*I sites of pET-30a(+), respectively, to generate pET-NbCK2α and pET-HvCK2α.

For GST pull-down of _XJ_TGB1 and _XJ_TGB3 proteins, the _XJ_TGB3 ORF was amplified via PCR and ligated in frame to the C terminus of the GST ORF at the *Bam*HI and *Xho*I restriction sites of the plasmid pGEX-KG (Guan and Dixon, 1991) to yield the pGEX-_XJ_TGB3 plasmid.

### Expression and purification of recombinant proteins from *Escherichia coli* BL21 cells

The pET plasmids designed for expression of the C-terminal His-tagged _XJ_TGB1 proteins, the _XJ_TGB1 protein mutants, and the HvCK2α and NbCK2α proteins were transformed into *E. coli* strain BL21(DE3) by standard procedures (Sambrook and Russell, 2001). Each transformant was grown overnight at 37 °C in 3ml of LB medium containing kanamycin (100 μg ml^–1^), transferred into 1 litre of fresh LB/kanamycin medium and grown to a density of 0.4 OD_600_. Recombinant protein expression was induced by the addition of 0.2mM isopropyl β-d-1-thiogalactopyranoside and the cells were shaken for 16 additional hours at 18 °C. Recombinant proteins were extracted from the bacteria and purified by Ni-affinity chromatography according to the manufacturer’s instructions (Bio-Rad) and evaluated by 12.5% SDS-PAGE.

### *In vitro* phosphorylation assays

Soluble cytoplasmic protein extracts of healthy *N. benthamiana* leaves were used for *in vitro* kinase assays according to the protocol described by Hung *et al.* (2014) and Vijayapalani *et al.* (2012). Phosphorylation reactions were performed with the *N. benthamiana* soluble protein extracts or with purified recombinant NbCK2α and HvCK2α subunits. Assays were performed with 1 μg of *N. benthamiana* soluble protein extracts, or 0.1 μg of recombinant CK2α, and 1 μg of purified _XJ_TGB1 protein or its mutants in a final volume of 10 μl of 25mM Tris/HCl (pH 7.4), 10mM MgCl_2_, and 1 μl [γ-^32^P]ATP or GTP (10 μCi, ~3000 Ci mmol^–1^; Perkin Elmer). Selected reactions were carried out in the presence or absence of heparin, and various concentrations of unlabelled ATP or GTP. Negative controls contained no TGB1 protein or 500 and 1000ng of bovine serum albumin. After incubation at 30 °C for 30min, the reactions were terminated by addition of 2.5 μl of 5× SDS buffer, and the samples were subjected to 12.5% SDS-PAGE. The gels were dried with a Model 583 Gel Dryer (Bio-Rad) and phosphorylated proteins were visualized by autoradiography.

### Mass spectrometry analysis

Phosphorylated _XJ_TGB1 and unphosphorylated _XJ_TGB1 proteins were digested with trypsin at 37 °C overnight. The digested peptides were then analysed by Q-Exactive liquid chromatography tandem mass spectrometry (LC-MS/MS) (Thermo Scientific) at the Mass Spectrometry Facility at China Agricultural University. The data were searched against the NCBI database using Mascot software with a 1% false discovery rate.

### Fluorescence and confocal microscopy

Green fluorescent protein (GFP) or red fluorescent protein (RFP) fluorescence in epidermal cells of *N. benthamiana* was observed with an Olympus confocol FV1000 microscope. GFP and RFP were excited at 488 or 546nm, respectively, with an argon laser. Images were recorded with an Olympus camera and processed using an Olympus Fluoview version 3.0 Viewer. In addition, cell-to-cell movement assays in epidermal cells of barley and *N. benthamiana* were observed with a BX53+DP72 fluorescence microscope (Olympus) and images were manipulated with the cellSens Entry programs.

### Electrophoretic mobility shift assays (EMSA)

RNAs for binding assays were transcribed *in vitro* in the presence of digoxigenin (DIG)–UTP (Roche) and the DIG-labelled transcripts were purified to remove the DNA templates (Wu *et al.*, 2013). Phosphorylation reactions were performed in a final volume of 5 μl containing phosphorylation assay buffer and purified NbCK2α, and different amounts of recombinant _XJ_TGB1 protein as described above. Negative controls consisted of samples lacking recombinant _XJ_TGB1 protein or 500 and 1000ng of bovine serum albumin. EMSA binding comparisons were performed by adding increasing amounts of protein to 50ng of purified RNA in binding buffer [50mM Tris/HCl (pH 7.5), 10mM MgCl_2_, 1mM EDTA] in a final volume of 20 μl. The binding reaction mixtures were incubated on ice for 30min and subjected to electrophoresis on 1% (w/v) non-denaturing agarose gels in 0.5× TBE buffer. RNA–protein complexes were transferred to a Hybond N^+^ nylon membrane (GE Healthcare) via a pump suction filter, and the RNA was cross-linked to the membrane by two 60 s cycles at 0.12 J in a Bio-Link crosslinker (Vilber Lourmat). Mobility shifts of the DIG-labelled RNAs were detected with DIG– alkaline phosphatasealkaline phosphatase Fab fragments (Roche), and the blots were developed with a 5-bromo-4-chloro-3-indolyl-phosphate/nitro blue tetrazolium chloride substrate solution (Amresco).

### GST pull-down

For co-expression of GST–_XJ_TGB3 with _XJ_TGB1–His or the _XJ_TGB1_T395A/T401A_–His fusion proteins, relevant plasmids were co-transformed into *E. coli* BL21(DE3). Cells were harvested by low-speed centrifugation and disrupted by vortexing in TB buffer [20mM Tris/HCl (pH 7.3), 500mM NaCl] in the presence of glass beads. The GST fusions and bound TGB proteins were purified by glutathione–Sepharose affinity chromatography and elution with glutathione (Pharmacia).

## Results

### Construction and sequence analysis of infectious clones of the BSMV Xinjiang strain

**Fig. 1.**
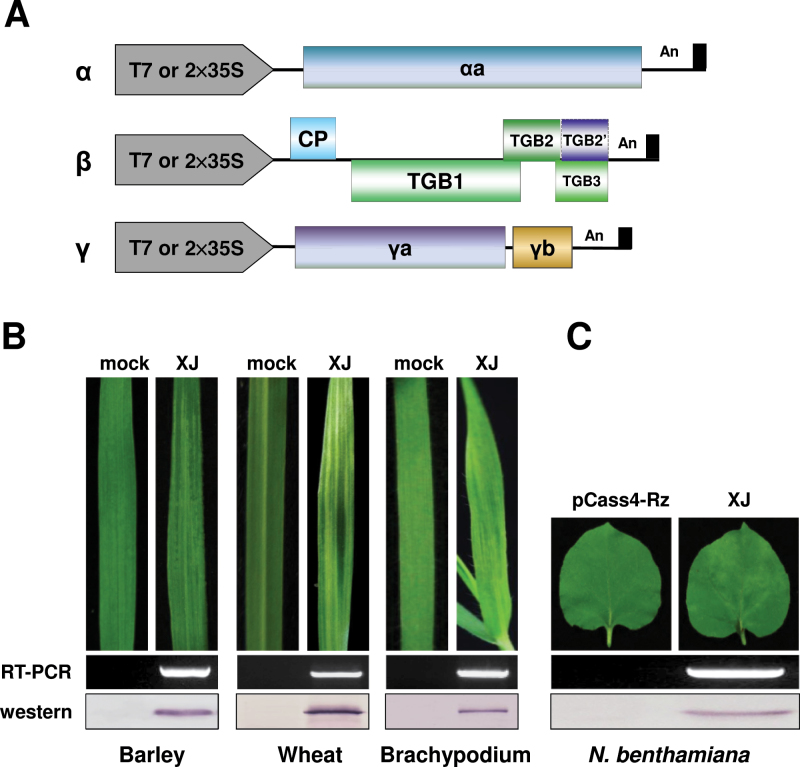
Diagram of _XJ_BSMV strain infectious clones and cereal and dicot host infectivity test results. (A) Illustration of _XJ_BSMV infectious clones under the T7 promoter or double cauliflower mosaic virus 35S promoter as described previously (Yuan *et al.*, 2011; Lee *et al.*, 2012). (B) Infectivity assays with *in vitro*-synthesized gRNAs of barley, wheat, and *B*. *distachyon* Bd21. (C) Agroinfiltration was used to initiate infections of *N. benthamiana*. Typical chlorotic stripes and mosaic symptoms (top) of BSMV appeared on emerging uninoculated leaves by 7–9 dpi. Upper uninoculated leaf tissue was harvested at 12 dpi, and the relative BSMV RNA and CP amounts were evaluated by RT-PCR (middle) and Western blotting with the antibody against BSMV CP (bottom). BSMV RNAγ was detected by RT-PCR with the primer pair BS-10/BS-32 (Supplementary Table S1). (This figure is available in colour at *JXB* online.)

Several BSMV field strains from China collected in our laboratory have broader host ranges than the more extensively studied _ND_BSMV and Type BSMV (_TY_BSMV) strains. To evaluate the diversity of the more virulent BSMV strains (Lee *et al.*, 2012), we constructed infectious clones of the _XJ_BSMV strain (Xie *et al.*, 1981) under the bacteriophage T7 or double cauliflower mosaic virus 35S promoters (Petty *et al.*, 1989; Yuan *et al.*, 2011) (Fig. 1A). The infectivity of *in vitro* transcripts synthesized from linearized pT7-α_XJ_, pT7-β_XJ_, and pT7-γ_XJ_ plasmids was tested by mechanical inoculation to barley, wheat, and *B*. *distachyon* Bd21. Inoculated plants consistently developed chlorotic stripes and mosaic symptoms typical of BSMV infections on upper uninoculated leaves by 6–7 dpi (Fig. 1B) and the efficiency of infectivity in barley, wheat, and *B*. *distachyon* Bd21 was 70–80, 80–90, and 50–60%, respectively (also see Supplementary Table S6). Agroinfiltration was used to initiate infections of *N. benthamiana* because only 10–30% of the plants became infected when using *in vitro* transcripts as inocula. *Agrobacterium* harbouring the plasmids pCa-α_XJ_, pCa-β_XJ_, and pCa-γ_XJ_ were infiltrated into the basal sides of the leaves. Newly emerging leaves developed mild mottling symptoms at 7–9 d after agroinfiltration (Fig. 1C), and the efficiency of infectivity was increased to approximate 90%. RT-PCR and Western blot analysis verified the infectivity of the _XJ_BSMV infectious clones in the monocot hosts (Fig. 1B) and in *N. benthamiana* (Fig. 1C).

For comparisons of _XJ_BSMV genomic variation with other published BSMV strains, the pT7-α_XJ_, pT7-β_XJ_ and pT7-γ_XJ_ cDNAs were sequenced. The results indicated that _XJ_RNAα (GenBank accession no. KJ746471), _XJ_RNAβ (GenBank accession no. KJ746472), and _XJ_RNAγ (GenBank accession no. KJ746473) consisted of 3789, 3222, and 2793 nt, respectively, and shared nucleotide identities of 95.2–99.8% (Supplementary Table S3, available at *JXB* online), 94.1–99.2% (Supplementary Table S4, available at *JXB* online), and 87.9–98.8% (Supplementary Table S5, available at *JXB* online) with RNAs α, β, and γ of other BSMV strains. These results suggest that substantial diversity may exist among BSMV strains.

### The _XJ_TGB1 protein is phosphorylated *in vitro* and *in vivo*

**Fig. 2.**
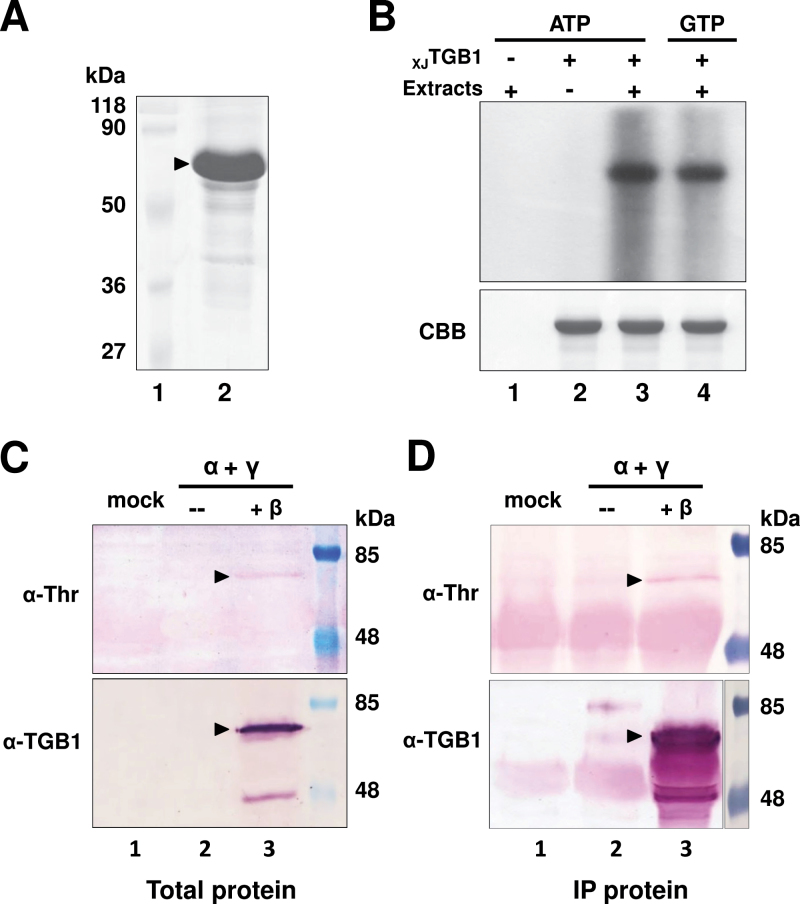
Phosphorylation of the _XJ_TGB1 protein *in vitro* and *in vivo*. (A) Coomasie Brilliant Blue (CBB) staining of recombinant _XJ_TGB1 protein purified from *E. coli* cells. Molecular weight markers (Fermentas) are indicated on the left side of the gel. (B) *In vitro* phosphorylation of purified _XJ_TGB1 protein by cellular kinases present in healthy *N. benthamiana* extracts in the absence or presence of [γ-^32^P]ATP or [γ-^32^P]GTP. After the phosphorylation reactions, the TGB1 proteins were separated by 12.5% SDS-PAGE and the incorporated radioactivity was analysed by autoradiography. Reaction mixtures lacking _XJ_TGB1 protein or *N. benthamiana* protein extracts served as negative controls. The CBB staining in the lower panel indicates that similar amounts of the _XJ_TGB1 protein were present in each *in vitro* phosphorylation reaction. (C) *In vivo* phosphorylation of _XJ_TGB1 protein in *N. benthamiana* by Western blotting with α-TGB1 polyclonal antibodies and α-threonine antibodies. A mock agroinfiltration lacking _XJ_RNAβ was used as a negative control and molecular weight markers (Thermo Scientific) were used to estimate the size of the _XJ_TGB1 protein. (D) *In vivo* phosphorylation of _XJ_TGB1 protein immunoprecipitated (IP) from *N. benthamiana* was analysed as in Fig. 2C. (This figure is available in colour at *JXB* online.)

In order to explore phosphorylation *in vitro*, the full-length _XJ_TGB1 protein was expressed as a C-terminal His-tagged fusion protein and purified to near homogeneity by Ni-affinity chromatography (Fig. 2A). A soluble protein kinase is known to be present in tobacco species (Hung *et al.*, 2014), and hence the purified _XJ_TGB1 protein was first assayed for phosphorylation using *N. benthamiana* protein extracts as a kinase source. The first control reaction containing [γ-^32^P]ATP and cytoplasmic extracts without the _XJ_TGB1 protein resulted in no distinct labelled products (Fig. 2B, lane 1). The corresponding control with the _XJ_TGB1 protein and [γ-^32^P]ATP alone suggested that the _XJ_TGB1 protein was not autophosphorylated *in vitro* (Fig. 2B, lane 2). In contrast, when both the cytoplasmic extracts and the _XJ_TGB1 protein were present, a radioactive phosphorylated product co-migrated with the _XJ_TGB1 protein (Fig. 2B, lane 3). These results provide evidence that the _XJ_TGB1 protein is phosphorylated *in vitro* with [γ-^32^P]ATP by a soluble kinase in the *N. benthamiana* extracts. The vast majority of protein kinases use ATP as an exclusive phosphate donor, whereas CK2 can effectively use either ATP or GTP (Matsushita *et al.*, 2000); hence we carried out phosphorylation comparisons with GTP to obtain clues about the identity of the kinase involved in _XJ_TGB1 phosphorylation. Autoradiography of the phosphorylated products revealed a single intense radiolabelled band when either [γ-^32^P]ATP or [γ-^32^P]GTP was used as a phosphoryl donor (Fig. 2B, lanes 3 and 4), implying that _XJ_TGB1 is phosphorylated by a CK2-like kinase in *N. benthamiana.*

To determine whether the _XJ_TGB1 protein is phosphorylated *in vivo*, _XJ_BSMV-infiltrated *N. benthamiana* leaves were harvested, concentrated by immunoprecipitation, and subjected to Western blot analysis with an anti-phosphothreonine (α-pT) antibody. The α-pT results revealed a labeleld protein with an electrophoretic mobility corresponding to the 58kDa _XJ_TGB1 protein (Fig. 2C and D, top, lane 3). Similar-sized bands were not observed in mock-inoculated leaves, or leaves infiltrated with only pCa-α_XJ_ and pCa-γ_XJ_ (Fig. 2C and D, top, lanes 1 and 2). Western blot analyses using the _XJ_TGB1 protein antibody confirmed that the α-pT-reacting protein co-migrated with _XJ_TGB1, and revealed a 45kDa immunospecific band that we suspect is a degradation product (Fig. 2C and D, bottom, lane 3). Collectively, these experiments provide convincing evidence that the _XJ_TGB1 protein is phosphorylated by a soluble CK2-like kinase *in vitro* and *in vivo*.

### CK2 is able to phosphorylate the _XJ_TGB1 protein

To predict potential _XJ_TGB1 protein phosphorylation kinases and sites, we analysed the _XJ_TGB1 protein sequence with the GPS 2.1 program tool (Xue *et al.*, 2011) and the Scansite Motif Scanner online server (http://scansite.mit.edu) (Obenauer, 2003). The GPS 2.1 program was set at a HIGH threshold (false-positive rates of 2% for Ser/Thr kinases) and the Scansite was set at a high stringency level to predict potential phosphorylation targets in the _XJ_TGB1 protein. According to the conserved CK2 phosphorylation site motif [(S/T)-X-X-(D/E); Meggio and Pinna, 2003], the GPS 2.1 predictions suggested that three residues in the _XJ_TGB1 protein (Ser-69, Thr-395, and Thr-401) are potential CK2 phosphorylation sites, whereas the Scanner only indicated that the Thr-401 site is phosphorylated by CK2 (Supplementary Fig. S1, available at *JXB* online). For comparison with ND18, the _XJ_TGB1 amino acids Ser-69, Thr-395, and Thr-401 correspond to the ND18 TGB1 (_ND_TGB1) Ser-70, Thr-396, and Thr-402 residues, respectively.

**Fig. 3.**
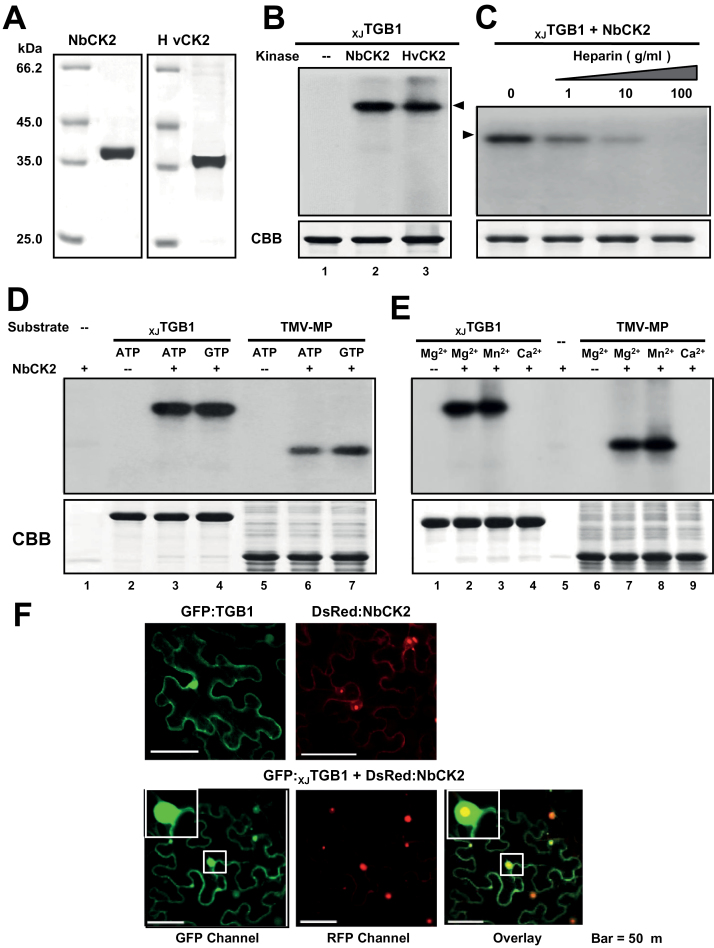
*In vitro* phosphorylation of _XJ_TGB1 protein by recombinant CK2 kinase. (A) SDS-PAGE analysis of NbCK2α and HvCK2α purified from *E. coli* BL21 cells. (B) *In vitro* phosphorylation of _XJ_TGB1 protein with the NbCK2α and HvCK2α recombinant proteins and negative controls lacking the kinases. (C) Effects of heparin on *in vitro* phosphorylation of _XJ_TGB1 protein. Phosphorylation levels were reduced with increasing amount of heparin. (D) Ability of NbCK2α to use both ATP and GTP as phosphate donors. (E) Divalent metal ion specificity of NbCK2α and the TMV-MP (P30) proteins. The CBB-stained proteins at the bottom of panels (B)–(E) are as indicated as in Fig. 2B. (F) Co-localization of the GFP:_XJ_TGB1 and DsRed:NbCK2α proteins in *N. benthamiana* leaf cells. Single localization of GFP:_XJ_TGB1 and DsRed:NbCK2α proteins are indicated at the top of the panels. Bars, 50 μm. (This figure is available in colour at *JXB* online.)

Based on the GTP results and phosphorylation predictions, we suspected that a CK2-like kinase might be responsible for phosphorylation of the _XJ_TGB1 protein. Therefore, the CK2α subunits from *N. benthamiana* (NbCK2α) and barley (HvCK2α) were cloned, and the C-terminal His-tagged fusion proteins were purified from *E. coli* cells (Fig. 3A). Subsequent *in vitro* phosphorylation assays revealed that the purified NbCK2α and HvCK2α proteins efficiently phosphorylated the recombinant _XJ_TGB1 protein in the presence of [γ-^32^P]ATP (Fig. 3B). CK2 is highly sensitive to heparin inhibition (Matsushita *et al.*, 2003), and our results confirmed that the levels of phosphorylation were reduced proportionally with increasing heparin concentrations (Fig. 3C). To further test the kinase specificity, we used NbCK2α to compare _XJ_TGB1 and TMV P30 MP (Ivanov *et al.*, 2003) protein phosphorylation in the presence of [γ-^32^P]ATP and [γ-^32^P]GTP (Fig. 3D). In both cases, the NbCK2α phosphorylation assays resulted in the presence of highly intense bands that co-migrated with the _XJ_TGB1 and P30 proteins, and the kinase exhibited similar activities in the presence of both ATP and GTP (Fig. 3D, lanes 3 and 4, and 6 and 7). Moreover, the absence of radioactive bands in reactions lacking the NbCK2α protein confirmed that the 58kDa _XJ_TGB1 and the P30 proteins are not autophosphorylated and the lack of radioactivity in the reactions without substrate proteins also indicated that the NbCK2α protein is not self-phosphorylated (Fig. 3D, lanes 2 and 5). In addition, tests were carried out in the presence of Mn^2+^, Mg^2+^, and Ca^2+^ to assess the cation specificity of CK2 phosphorylation (Niefind *et al.*, 1999). These results revealed that NbCK2α exhibited similar phosphorylation intensities for _XJ_TGB1 and P30 in the presence of either Mn^2+^ or Mg^2+^ (Fig. 3E, lanes 2 and 3, and 7 and 8), and that ^32^P incorporation was negligible in reactions containing Ca^2+^ (Fig. 3E, lanes 4 and 9). All of the *in vitro* phosphorylation data with the TGB1 protein were consistent with several published biochemical properties of CK2 (Ivanov *et al.*, 2003; Hung *et al.*, 2014).

Furthermore, to explore the potential co-localization of the _XJ_TGB1 protein and NbCK2α *in planta*, we conducted co-expression experiments with GFP:_XJ_TGB1 and DsRed:NbCK2α fusion proteins in *N. benthamiana* cells. Confocal microscopy revealed that GFP:_XJ_TGB1 was distributed in both the cytoplasm and nucleus, whereas DsRed:NbCK2α was present primarily in the nucleus. The co-localization of the two proteins indicated that the _XJ_TGB1 protein and NbCK2α interact in both the nucleus and cytoplasm in some manner (Fig. 3F, overlay channel). Taken together, the presented data demonstrate that XJTGB1 is phosphorylated by CK2 *in vitro* and *in planta*.

### Thr-401 is the major _XJ_TGB1 protein site for CK2 phosphorylation

**Fig. 4.**
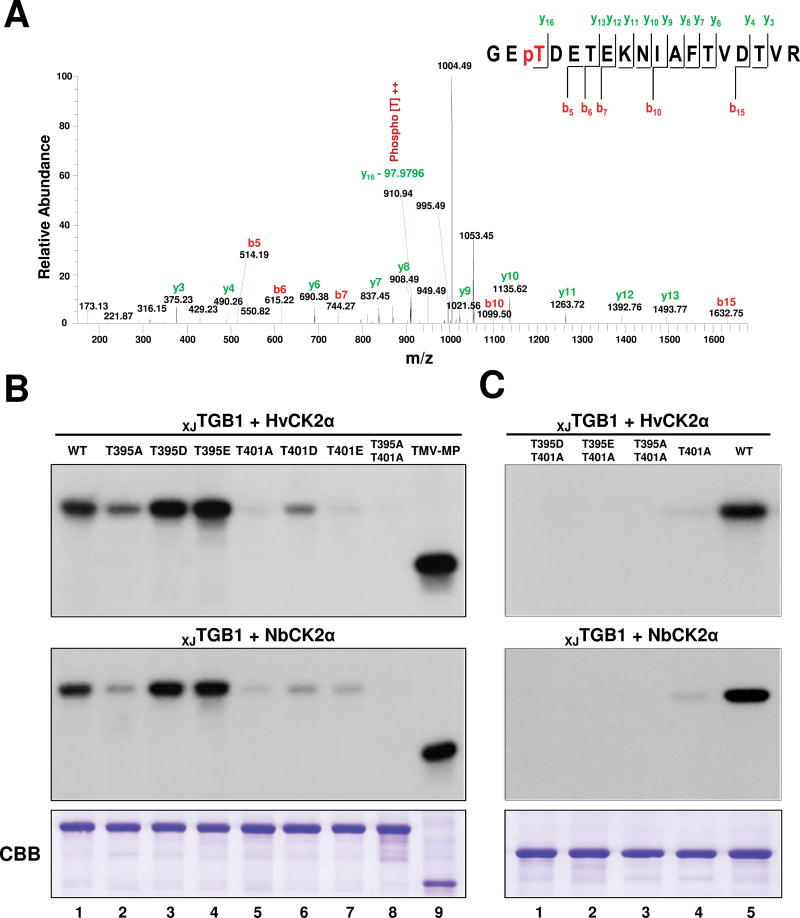
Thr-401 is the major _XJ_TGB1 protein site for CK2 phosphorylation. (A) LC-MS/MS analysis of _XJ_TGB1 protein phosphorylation by NbCK2α. The absence of phosphoric acid (97.9769Da) on the y_16_ ion fragment demonstrates that Thr-401 is a phosphorylation site for CK2 kinase. (B) Identification of the phosphorylation sites in _XJ_TGB1 protein mutants by *in vitro* phosphorylation with HvCK2α and NbCK2α. The radioactive intensities of the _XJ_TGB1 protein and its phosphorylation mutants indicate the extent of radiolabelling with [γ-^32^P]ATP. CBB-stained proteins at the bottom of the panels (B) and (C) are as indicated in Fig. 2B. (C) Phosphorylation comparisons of selected _XJ_TGB1 protein mutants with wt _XJ_TGB1 protein to confirm that Thr-401 is the major phosphorylated residue. (This figure is available in colour at *JXB* online.)

To identify the phosphorylation sites of CK2, the purified _XJ_TGB1 protein was phosphorylated by NbCK2α *in vitro* with unlabelled ATP, and the gel-purified phosphorylated and unphosphorylated _XJ_TGB1 proteins were separated by PAGE and digested with trypsin. The trypsin digestion products were analysed by Q-Exactive LC-MS/MS. The analysis showed that 87.9% of the TGB1 protein amino acid sequence was covered, and revealed that the phosphorylated and unphosphorylated proteins differed in a unique monophosphorylated peptide (^399^GE**T**DETEKNIAFTVDTVR^416^) with a 2103.9362 *m*/*z* peak corresponding to a neutral precursor ion lacking phosphoric acid (97.9769Da). Based on the observed masses of the phosphorylated and unphosphorylated y_16_ fragment ions (Fig. 4A), we conclude that the phosphorylated _XJ_TGB1 residue is located at Thr-401.

To verify the GPS 2.1 and Scanner predictions (Supplementary Fig. S1) and LC-MS/MS analysis of _XJ_TGB1 protein, we replaced one or both of the Thr-395 and Thr-401 residues with alanine residues to produce _XJ_TGB1_T395A_, _XJ_TGB1_T401A_, and _XJ_TGB1_T395A/T401A_ phosphorylation-deficient mutants. To mimic the phosphorylation state of the _XJ_TGB1 protein, Thr-395 and Thr-401 residues were substituted with aspartic acid (D) or glutamic acid (E) residues to produce the _XJ_TGB1_T395D_, _XJ_TGB1_T395E_, _XJ_TGB1_T401D_, and _XJ_TGB1_T401E_ mutants. *In vitro* phosphorylation comparisons of the wild-type (wt) _XJ_TGB1 protein, and the Thr-395 and Thr-401 mutants were performed with HvCK2α and NbCK2α, respectively. Compared with the wt _XJ_TGB1 protein (Fig. 4B, lane 1), the phosphorylation level of the _XJ_TGB1_T395A_ mutant protein was reduced partially (Fig. 4B, lane 2). However, both the _XJ_TGB1_T395D_ and the _XJ_TGB1_T395E_ mutant proteins incorporated slightly larger amounts of ^32^P label than the wt _XJ_TGB1 protein (Fig. 4B, compare lane 1 with lanes 3 and 4), suggesting that the positive charges imparted by the aspartic acid and glutamic acid residues increased the kinase efficiency. As anticipated, the phosphorylation intensities of the _XJ_TGB1_T401A_ (Fig. 4B, lane 5) and _XJ_TGB1_T401E_ (Fig. 4B, lane 7) proteins only showed faint shadows. We also observed similar reductions during incorporation into the _XJ_TGB1_T401D_ protein (Fig. 4B, lane 6). We believe that Thr-395 may have been phosphorylated, and this is supported by negligible incorporation into the three double mutants (Fig 4B, lane 8; Fig. 4C, lanes 1–3). Thus, our interpretation of these results is that Thr-401 is a major phosphorylation site and that Thr-395 is a minor phosphorylation target.

Based on the data above and previous analyses of 308 CK2 phosphorylation sites for other proteins (Meggio and Pinna, 2003), we propose that Thr-395 is a docking site for CK2 because phosphorylation of this residue enhanced kinase activity at Thr-401. To evaluate this hypothesis, we used a double mutant, _XJ_TGB1_T395A/T401A_, for phosphorylation. The results revealed extremely low, if any, ^32^P incorporation into other TGB1 residues (Fig. 4B, lane 8; Fig 4C, lane 3). To determine whether there was ‘off-site’ targeting of other residues within _XJ_TGB1 at the proposed Thr-395 docking site, we engineered the _XJ_TGB1_T395D/T401A_ and _XJ_TGB1_T395E/T401A_ double mutants, which could not be phosphorylated at Thr-401, and both double mutants had negligible levels of ^32^P incorporation (Fig. 4C, lanes 1 and 2). In summary, these results support the hypothesis that Thr-395 functions as a docking residue and that Thr-401 is the major phosphorylation site within the kinase motif of the _XJ_TGB1 protein.

### Mutations of the _XJ_TGB1 protein phosphorylation site affect BSMV local and systemic infections of dicots and monocots

**Fig. 5.**
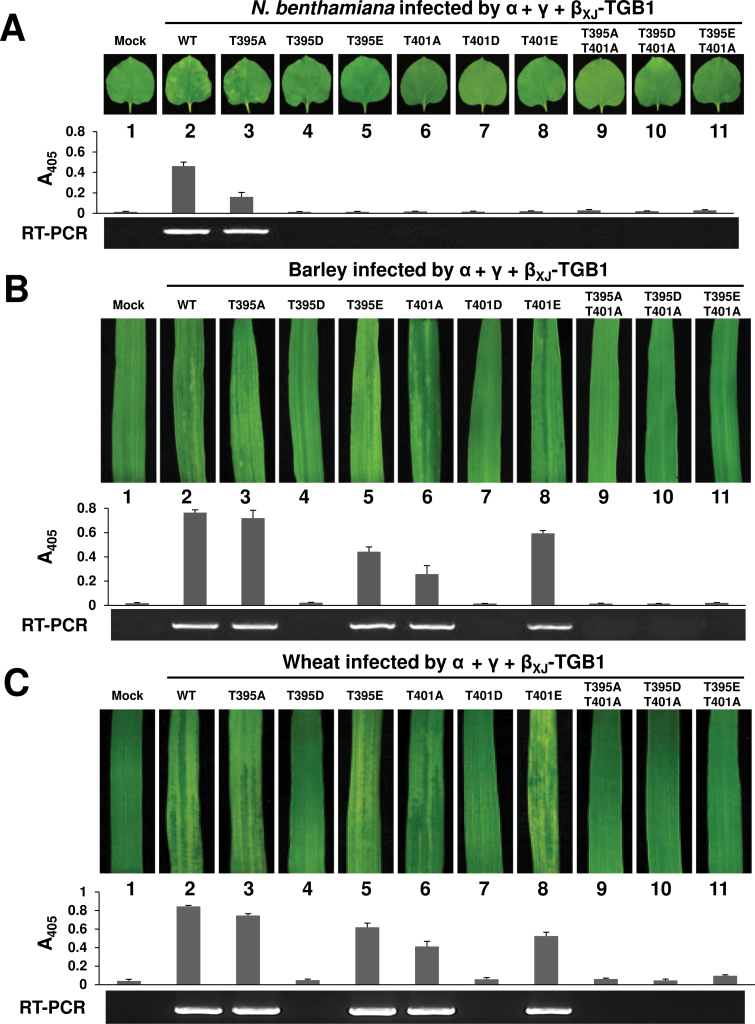
Mutants affecting phosphorylation of the _XJ_TGB1 protein have host-specific effects on systemic infectivity. (A) Symptoms of *N. benthamiana* elicited after infiltration with an *Agrobacterium* mixture harbouring pCa-α_XJ_, pCa-γ_XJ_, and pCa-β_XJ_ or its phosphorylation site mutants. Upper uninfiltrated leaf tissues were harvested and photographed at 10 dpi (top). CP ELISA (middle) and RNAγ RT-PCR amplification (bottom) were monitored to estimate the infectivity levels. (B, C) Systemic symptoms appearing in barley (B) and wheat (C) after inoculation with pT7-α_XJ_ and pT7-γ_XJ_ *in vitro* transcripts mixed with pT7-β_XJ_ and various phosphorylation mutant transcripts. Leaves were photographed at 14 dpi (top) and all experiments were repeated three times. (This figure is available in colour at *JXB* online.)

To determine whether the _XJ_TGB1 mutants affected systemic movement in *N. benthamiana*, leaves were infiltrated with *Agrobacterium* harbouring the pCa-α_XJ_, wt pCa-β_XJ_, or individual TGB1 mutant derivatives, and the pCa-γ_XJ_ clones. Three independent experiments revealed that only wt _XJ_TGB1 and the β_XJ_-TGB1_T395A_ mutant, which exhibited slightly lower phosphorylation levels than the wt _XJ_TGB1 protein, were able to establish systemic infections at 10 dpi (Fig. 5A, lanes 2 and 3). None of the remaining infiltrations containing single or double _XJ_TGB1 mutants developed mosaic symptoms or invaded the upper uninoculated leaves as assessed by the absence of CP accumulation (Fig. 5A, lanes 4–11).

For systemic infectivity on monocots, barley and wheat leaves were co-inoculated with *in vitro* transcripts of pT7-α_XJ_, pT7-β_XJ_ (wt or mutant derivatives), and pT7-γ_XJ_. Visual observations, ELISA and RT-PCR analyses demonstrated that the barley and wheat plants had similar systemic infections on upper uninoculated leaves at 14 dpi after wt and mutant _XJ_TGB1 inoculations (Fig. 5B, C). In the case of β_XJ_-TGB1_T395A_, a milder infection phenotype was noted on the systemically infected cereal leaves, compared with those of the wt β_XJ_ infections (Fig. 5B, C, lanes 2 and 3), but the aspartic acid mutant β_XJ_-TGB1_T395D_ was unable to infect the plants systemically (Fig. 5B, C, lane 4). In contrast, when inoculated with the β_XJ_-TGB1_T395E_ mutant, which more effectively mimics phosphorylation of threonine residues, the cereal plants exhibited CP accumulation levels that were similar to wt BSMV (Fig. 5B, C, lane 5). Moreover, the β_XJ_-TGB1_T401E_ mutant elicited systemic infections, albeit with slightly lower CP levels (Fig. 5B, C, lane 8), but the levels were higher than those with β_XJ_-TGB1_T401A_ (Supplementary Table S6, available at *JXB* online). However, the β_XJ_-TGB1_T395A/T401A_, β_XJ_-TGB1_T395D/T401A_, and β_XJ_-TGB1_T395E/T401A_ double mutants were unable to infect either barley or wheat (Fig. 5B, lanes 9–11).

Taken together, these results demonstrated that the _XJ_TGB1 phosphorylation site mutations generally reduced the infection efficiencies in dicots and monocots, and that the mutants had more dramatically compromised systemic movement phenotypes in *N. benthamiana*. To summarize, the T395A, T395E, T401A, and T401E mutants exhibited systemic movement in barley and wheat, whereas only the T395A mutant is able to establish systemic infections in *N. benthamiana*.

We next conducted experiments to evaluate the cell-to-cell movement profiles of the Thr-395 and Thr-401 mutants in *N. benthamiana* and barley. For this purpose, *N. benthamiana* leaves were infiltrated with *Agrobacterium* containing pCa-α_XJ_, pCa-β_XJ_ and its mutant derivatives, and a pCa-γ_XJ:GFP_ construct that harbours a γb:GFP reporter gene to assess cell-to-cell movement. In barley, leaves were co-inoculated with T7 transcripts of the RNAα, RNAβ derivatives, and RNAγ-γ_XJ:GFP_ transcripts. Epidermal cells of the leaves were observed by confocal microscopy at 3 dpi and compared with inoculated controls lacking the RNAβ.

**Fig. 6.**
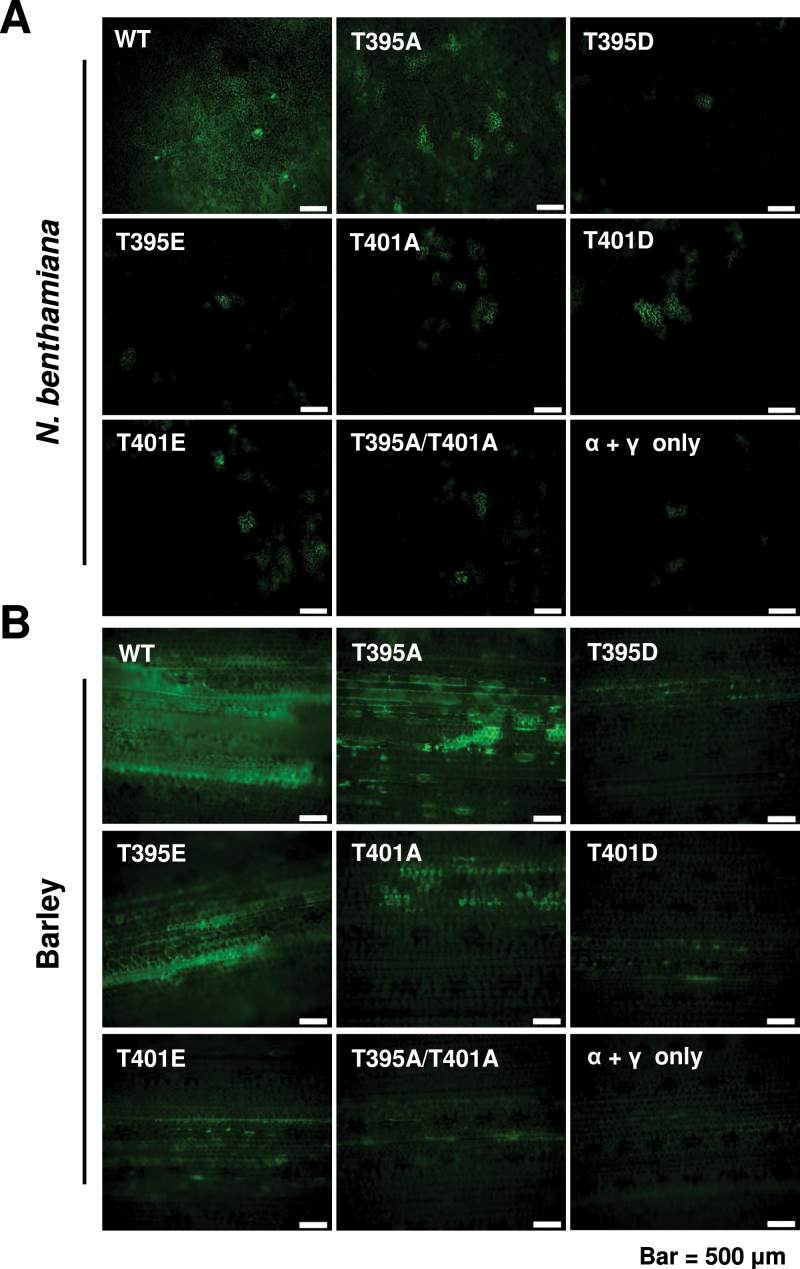
Effects of _XJ_TGB1 protein phosphorylation on _XJ_BSMV cell-to-cell movement in *N. benthamiana* and barley. (A) Fluorescence in *N. benthamiana* leaves at 3 dpi with an *Agrobacterium* mixture of pCa-α_ND_, pCa-γ_ND:GFP_, and pCa-β_XJ_ or the pCa-β_XJ_ mutant derivatives. The total bacterial concentrations for infiltration were OD_600_ of 0.08. (B) Fluorescence in barley leaves at 3 dpi with *in vitro* transcripts of RNAα and RNAγ:GFP plus wt _XJ_RNAβ or the _XJ_RNAβ phosphorylation site mutant derivatives. Bars represent 500 μm. (This figure is available in colour at *JXB* online.)

The localized movement in infiltrated *N. benthamiana* leaves generally reflected the systemic infection phenotypes elicited by the mutants (Fig. 6A). In *N. benthamiana*, the wt β_XJ_ and β_XJ_-TGB1_T395A_ mutant both exhibited cell-to-cell movement encompassing several cells at 3 dpi (Fig. 6A), as expected due to their ability to elicit systemic infections. The remaining mutants usually developed fluorescence in a single cell or rarely in two to three adjacent cells (Fig. 6A). Hence, the localized movements of the mutants correlated reasonably well with their systemic movement patterns in *N. benthamiana*. In barley leaves, most of the fluorescence at 3 dpi appeared in mesophyll cells, but in this case, the virus had to traverse only two to three cell layers before encountering the closely aligned parallel vasculature (Fig. 6B). Hence, the β_XJ_-TGB1_T395A_, β_XJ_-TGB1_T395E_, and β_XJ_-TGB1_T401A_ mutants that established systemic infections in barley and wheat needed to negotiate only a limited number of mesophyll cells to reach the vascular elements for systemic spread, whereas movement through a larger number of cells was required to reach the dicot vasculature. The other mutants, β_XJ_-TGB1_T395D_, β_XJ_-TGB1_T401D_, and β_XJ_-TGB1_T395A/T401A_, exhibited more limited cell-to-cell movement compared with β_XJ_-TGB1_T395A_, β_XJ_-TGB1_T395E_, and β_XJ_-TGB1_T401A_, but could spread to a few adjacent cells. However, these three mutants were unable to invade the upper cereal leaves. Hence, these results demonstrate that phosphorylation activities at Thr-395 and Thr-401 differentially affecte systemic movement in monocot versus dicot hosts and suggest that at least some of the host-specific results may be a consequence of the vasculature architecture of these hosts.

### Phosphorylation promotes virus infection of _XJ_BSMV by enhancing TGB1 and TGB3 protein interactions

Previous studies have shown that BSMV spreads from cell to cell through the co-ordinated actions of TGB proteins, which co-localize at the cell wall in close association with PD, during cell-to-cell movement in monocots and dicots (Lim *et al.*, 2008). Our results shown above demonstrated that interference with CK2 phosphorylation at the _XJ_TGB1 Thr-395 and Thr-401 sites affected _XJ_BSMV local and systemic movement. TGB1 is a multifunctional protein that engages in homologous interactions and formation of a ribonucleoprotein complex containing viral genomic and subgenomic RNAs (Lawrence and Jackson, 2001). Therefore, we used three approaches to identify _XJ_TGB1 protein functions affected by CK2 phosphorylation.

**Fig. 7.**
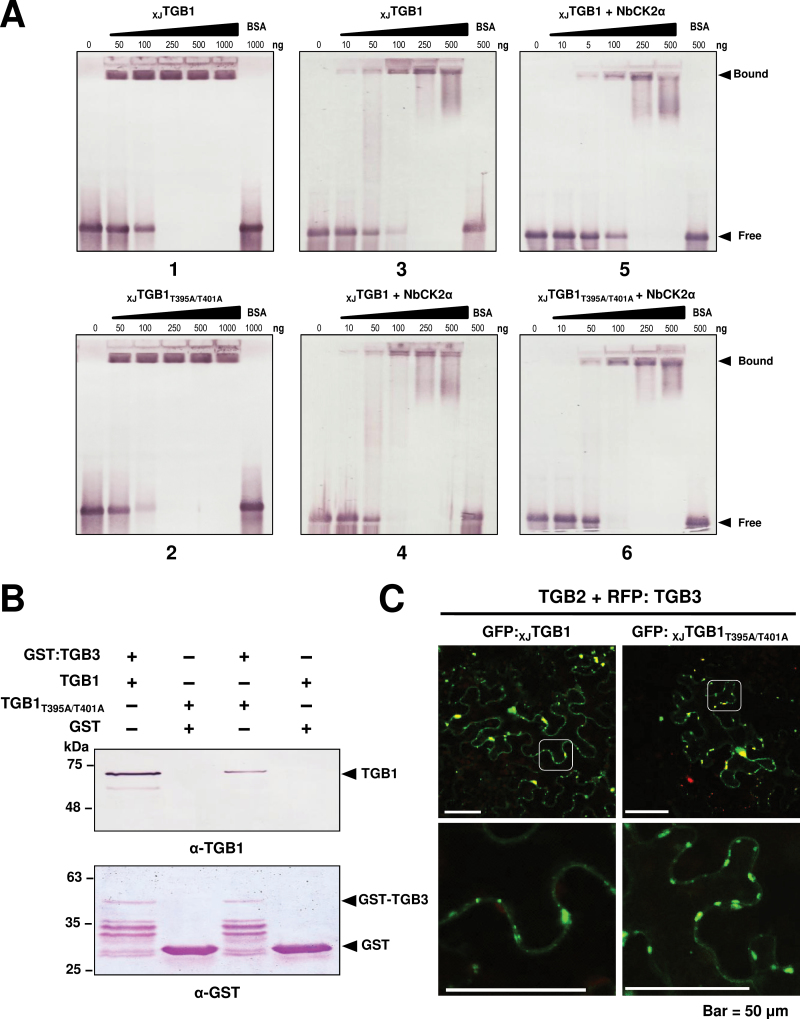
Effect of _XJ_TGB1 protein phosphorylation mutants on its functions. (A) Comparison of RNA binding by the phosphorylated native _XJ_TGB1 and double-mutant _XJ_TGB1_T395A/T401A_ proteins. (B) GST affinity chromatography comparisons of the _XJ_TGB1 and double-mutant _XJ_TGB1_T395A/T401A_ proteins with the GST:_XJ_TGB3 protein. The concentrations of the TGB proteins were similar in the experiments, but the _XJ_TGB1_T395A/T401A_ protein had approximate 40% TGB3 protein-binding efficiency compared with the wt _XJ_TGB1 protein. The illustrated binding result is typical of three independent experiments. (C) Co-localization of TGB proteins. Confocal laser-scanning microscopy observation of *N. benthamiana* leaf epidermal cells co-infiltrated with mixtures of *Agrobacterium* harbouring GFP:_XJ_TGB1 or the GFP:_XJ_TGB1_T395A/T401A_ mutant derivatives and the pGD-TGB2 and RFP:TGB3 plasmids. Bars, 50 μm. (This figure is available in colour at *JXB* online.)

To determine whether the RNA-binding affinity of the _XJ_TGB1 protein changed upon phosphorylation, we first used purified wt _XJ_TGB1 and the double mutant _XJ_TGB1_T395A/T401A_ proteins in EMSA with DIG-labelled RNA transcripts (Fig. 7A). The results clearly showed that both proteins bound almost all of the available RNA at 250ng, indicating that the mutant protein did not affect RNA-binding activities (Fig. 7A, panels 1 and 2). Next, to determine phosphorylation effects directly, the _XJ_TGB1 protein was incubated in phosphorylation assay buffer containing unlabelled ATP, with and without the addition of purified NbCK2α, and EMSA assays were performed to compare the abilities of the non-phosphorylated and phosphorylated _XJ_TGB1 proteins to bind the labelled RNA transcripts (Fig. 7A, panels 3 and 4). In addition, the RNA-binding activities of the phosphorylated wt _XJ_TGB1 and mutant _XJ_TGB1_T395A/T401A_ proteins were compared, but the _XJ_TGB1_T395A/T401A_ protein was found to retain almost the same level of RNA binding as the wt _XJ_TGB1 protein (Fig. 7A, panels 5 and 6). Hence, phosphorylation appeared to have little, if any, effect on the RNA-binding activities of the _XJ_TGB1 protein.

To evaluate the possible role of phosphorylation in heterologous interactions of _XJ_TGB1 and _XJ_TGB3 proteins, experiments were carried out with His-tagged _XJ_TGB1 and its mutant _XJ_TGB1_T395A/T401A_ fusion proteins in co-expressions with the GST:_XJ_TGB3 protein in *E. coli* BL21 cells. Both _XJ_TGB1 and _XJ_TGB1_T395A/T401A_ proteins were retained to some extent on the affinity columns by the GST:_XJ_TGB3 protein (Fig. 7B), but our three experiments consistently showed that the _XJ_TGB1_T395A/T401A_ protein bound the TGB3 protein approximate 40% less effectively than the _XJ_TGB1 protein. These results thus suggested that the mutant _XJ_TGB1_T395A/T401A_ may weaken TGB1:TGB3 protein interactions and result in impaired cell-to-cell movement functions of _XJ_BSMV.

In additional attempts to ascertain whether the compromised TGB1:TGB3 protein interactions or CK2 phosphorylation affected localization of TGB proteins in plant cells, co-localization assays were performed by transient expression of the three TGB proteins via agroinfiltration in *N. benthamiana*, and GFP and RFP localizations were evaluated at 2 dpi by confocal laser-scanning microscopy. The results revealed that TGB2 and TGB3, the _XJ_TGB1_T395A/T401A_ protein, and the wt _XJ_TGB1 protein had similar TGB localization patterns (Fig. 7C). Taken together, we conclude that phosphorylation promotes _XJ_BSMV infection by enhancing the interactions between the _XJ_TGB1 and _XJ_TGB3 proteins, although this effect was not sufficient to substantially alter the TGB localization patterns visible by confocal microscopy.

## Discussion

Reversible phosphorylation and dephosphorylation of proteins have regulatory roles in a wide range of cellular processes, including cell signalling transduction (Moreno-Romero *et al.*, 2011), protein subcellular localization (Nardozzi *et al.*, 2010), and protein–protein (Trott *et al.*, 2001) and protein–nucleic acid interactions (Schuck *et al.*, 2013). Numerous proteins with distinct phosphorylation sites have been investigated, and protein kinases affecting a wide range of cellular responses have been characterized (Peck, 2006; Ubersax and Ferrell, 2007; Bond *et al.*, 2011; Dissmeyer and Schnittger, 2011). A growing body of evidence now shows that viral proteins with different functions are phosphorylated by various protein kinases during infection. These include CK2, PKA, PKC, and CK1 protein kinases (Lee and Lucas, 2001; Link *et al.*, 2011; Makarov *et al.*, 2012; Hung *et al.*, 2014), and among these kinases, CK2 phosphorylation effects on infectivity have been most extensively studied.

Protein kinase CK2, a conserved Ser/Thr kinase existing in almost all eukaryotes, phosphorylates proteins with a consensus phosphorylation site motif (S/T-D/E-X-E/D, where X is any residue) (Ubersax and Ferrell, 2007). Increasing evidence indicates that CK2 protein phosphorylation has important roles in plant growth and development (Mulekar *et al.*, 2012), and that CK2 also regulates virus infection processes, including virion assembly, cell-to-cell and long-distance movement, and interactions between viral proteins and other host proteins (Jakubiec and Jupin, 2007; Nardozzi *et al.*, 2010). In addition to the MP phosphorylation effects mentioned in the Introduction, phosphorylation of cucumber mosaic virus, cucumber necrosis virus, and turnip yellow mosaic virus RdRp has substantial effects on virus replication (Kim *et al.*, 2002; Shapka *et al.*, 2005; Stork *et al.*, 2005; Jakubiec *et al.*, 2006; Jakubiec and Jupin, 2007). Ser/Thr phosphorylation has also been suggested to affect the CP functions of several plant viruses. For example, phosphorylation of potato virus A (PVA) CP by host CK2 inhibits viral RNA binding *in vitro*, and mutation of a major phosphorylation CP site generates a PVA variant that is defective in cell-to-cell and long-distance movement (Ivanov *et al.*, 2001, 2003). In addition, phosphorylation of the cauliflower mosaic virus CP precursor at several sites by CK2 is important for virus infectivity and symptom development (Chapdelaine *et al.*, 2002). Phosphorylation of the bamboo mosaic virus CP by CK2 also regulates cell-to-cell movement by modulating RNA binding (Hung *et al.*, 2014).

Although phosphorylation of the PSLV N-terminal portion of the TGB1 protein has been reported (Makarov *et al.*, 2012), but the results were not extended to evaluate the roles of phosphorylation in PSLV movement processes. Our results now demonstrate that the BSMV TGB1 protein is phosphorylated by CK2 *in vitro* and *in planta*, and that the phosphorylation events affect virus movement. Although the prediction programs we used suggest that the _XJ_TGB1Thr-395 residue in the ^395^ TDYD^398^ site is more likely to be a conserved CK2 phosphorylation site than the Thr-401 (^401^ TDET^404^) site (Meggio and Pinna, 2003), it is noteworthy that Thr-401 localizes within an acidic residue-rich region (^399^GETDETEK^**406**^) that may be more favourable for phosphorylation (Battistutta *et al.*, 2000; Riera *et al.*, 2001) than the Thr-395 residue. Thus, based on the *in vitro* phosphorylation assays of the TGB1 mutant derivatives, as well as results derived from LC-MS/MS analysis, we conclude that Thr-401 is the major TGB1 phosphorylation site and that Thr-395 functions as a CK2 docking site and has a more limited phosphorylation role. To the best of our knowledge, this is the first report showing that a plant viral protein, which can be phosphorylated by CK2, has a CK2 docking site adjacent to the phosphorylation site.

To determine the effects of _XJ_TGB1 Thr-395 and Thr-401 phosphorylation on virulence, point mutations were introduced into the _XJ_BSMV clone. Infectivity results in the monocot and dicot hosts revealed that the mutant derivatives differed in their systemic movement phenotypes. For example, the T395A mutant was the only derivative able to infect *N. benthamiana*, barley, and wheat systemically, but the T395E, T401A, and T401E mutants also systemically infected the monocot hosts (Fig. 5). Our results suggest that the mutants may be compromised by partial disruption of phosphorylation and dephosphorylation dynamics in TGB1 in ways that contribute to diminished cell-to-cell movement. However, the amino acid structures of the T395A, T395D, T395E, T401D, and T401E substitutions are not entirely consistent with this simplistic model, as it is obvious that the substituted amino acids differ in the sizes of their side chains and their charges. Moreover, we cannot exclude the possibility that ‘off-site’ phosphorylation of Thr-395 or phosphorylation by kinases other than CK2 may be elicited by the substitutions and that these events may contribute to protein modifications that affect local and vascular movement.

Hordeivirus TGB1 proteins are multifunctional and contain two positively charged regions rich in lysine (K) and arginine (R) residues at the N-terminal half of the protein and a C-terminal region consisting of seven conserved motifs (I, IA, II, III, IV, V, and VI) (Jackson *et al.*, 2009). The Thr-395 and Thr-401 sites are located between domain IV and V of the TGB1 protein and are not included in the most highly conserved regions. The hordeivirus TGB1 proteins have multiple ssRNA- and dsRNA-binding sites, and hence mutagenesis of single or closely associated _XJ_TGB1 protein sites may not have obvious effects on RNA-binding activities *in vitro*.

We have shown previously that the BSMV TGB1 protein is the major protein component of ribonucleoprotein complexes involved in BSMV cell-to-cell movement. TGB1 also participates in interactions of TGB1 and TGB3 proteins during intra- and intercellular virus movement, and functions in TGB1:TGB3 interactions that recruit the TGB2 protein during transport through PD to adjacent cells (Jackson *et al.*, 2009). These interactions are critical for movement because TGB3 serves as a bridge to direct TGB co-localization at the cell wall and to establish close associations with the PD (Lim *et al.*, 2009). Even though both CK2 phosphorylation sites (T395A/T401A) in _XJ_TGB1 were mutated simultaneously, the mutant TGB1 protein did not elicit obvious changes in subcellular localization patterns (Fig. 7C). However, the _XJ_TGB1_T395A/T401A_ protein did reduce binding affinity to the TGB3 protein compared with the wt TGB1 protein. Therefore, our results provide evidence that CK2 phosphorylation of TGB1 affects BSMV movement by modulating TGB1:TGB3 protein interactions.

In summary, our results shown here provide evidence showing that phosphorylation of the BSMV _XJ_TGB1 protein by CK2 at C-terminal residues affects cell-to-cell movement and the systemic infection phenotype. Moreover, the mutant results are compatible with a model whereby modulation of TGB1:TGB3 interactions contribute to phenotypic differences with in BSMV movement in monocot and dicot hosts. Compared with the TGB1 proteins of other hordeiviruses (Supplementary Fig. S2A, available at *JXB* online), Thr-401 but not Thr-395 is conserved in PSLV TGB1 and BSMV TGB1. This implies that the proposed docking site function of Thr-395 may be unique for BSMV phosphorylation. Furthermore, multiple sequence alignments of TGB1 proteins (Supplementary Fig. S2B) showed that the Thr-395 and Thr-401 sites are highly conserved among six sequenced BSMV strains, suggesting that phosphorylation of the TGB1 protein is required during infection of all BSMV strains.

## Supplementary data

Supplementary data are available at *JXB* online.

Supplementary Fig. S1. Phosphorylation predictions of the _XJ_TGB1 protein by the GPS 2.1 program (A) and the Scansite Motif Scanner online server (B).

Supplementary Fig. S2. Alignment of the TGB1 proteins of the hordeiviruses (A) and among six sequenced BSMV strains (B).

Supplementary Table S1. Primers used in construction and analysis of biologically active BSMV Xinjiang cDNA clones.

Supplementary Table S2. Primers used for site-specific mutagenesis of Xinjiang RNAβ clones.

Supplementary Table S3. Sequence alignment of Xinjiang strain RNAα with different BSMV strains.

Supplementary Table S4. Sequence alignment of Xinjiang strain RNAβ with different BSMV strains.

Supplementary Table S5. Sequence alignment of Xinjiang strain RNAγ with different BSMV strains.

Supplementary Table S6. Systemic infectivity efficiency of _XJ_BSMV TGB1 phosphorylation mutants on dicot and monocot hosts.

Supplementary Data

## Abbreviations:

BSMV: barley stripe mosaic virus
CP: coat protein
DIG: digoxygenin
dpi: d post-inoculation
EMSA: electrophoretic mobility shift assays
GFP: green fluorescent protein
LC-MS/MS: liquid chromatography tandem mass spectrometry
MP: movement protein
ORF: open reading frame
PD: plasmodesmata
PSLV: Poa semilatent virus
RdRp: RNA-dependent RNA polymerase
RFP: red fluorescent protein
RT-PCR: reverse transcription-PCR
TGB: triple gene block
TMV: tobacco mosaic virus.
