# Supplementary material for: Phosphorylation of TGB1 by protein kinase CK2 promotes barley stripe mosaic virus movement in monocots and dicots
Source: J Exp Bot. 2015 May 21;66(15):4733–47. doi: 10.1093/jxb/erv237 (PMC4507770; doi:10.1093/jxb/erv237)
Supplement: Supplementary Data [file supp_66_15_4733__index.html]

Phosphorylation of TGB1 by protein kinase CK2 promotes barley stripe mosaic virus movement in monocots and dicots — Phosphorylation of TGB1 by protein kinase CK2 promotes barley stripe mosaic virus movement in monocots and dicots — Supplementary Data 

# Phosphorylation of TGB1 by protein kinase CK2 promotes barley stripe mosaic virus movement in monocots and dicots

## Supplementary Data

Data files

**Files in this Data Supplement:**

- Supplementary Data - Supplementary Data
