# Supplementary material for: Phosphorylation of TGB1 by protein kinase CK2 promotes barley stripe mosaic virus movement in monocots and dicots
Source: J Exp Bot. 2015 May 21;66(15):4733–47. doi: 10.1093/jxb/erv237 (PMC4507770; doi:10.1093/jxb/erv237)
Supplement: Supplementary Data [file supp_erv237_jexbot148064_file001.pdf]

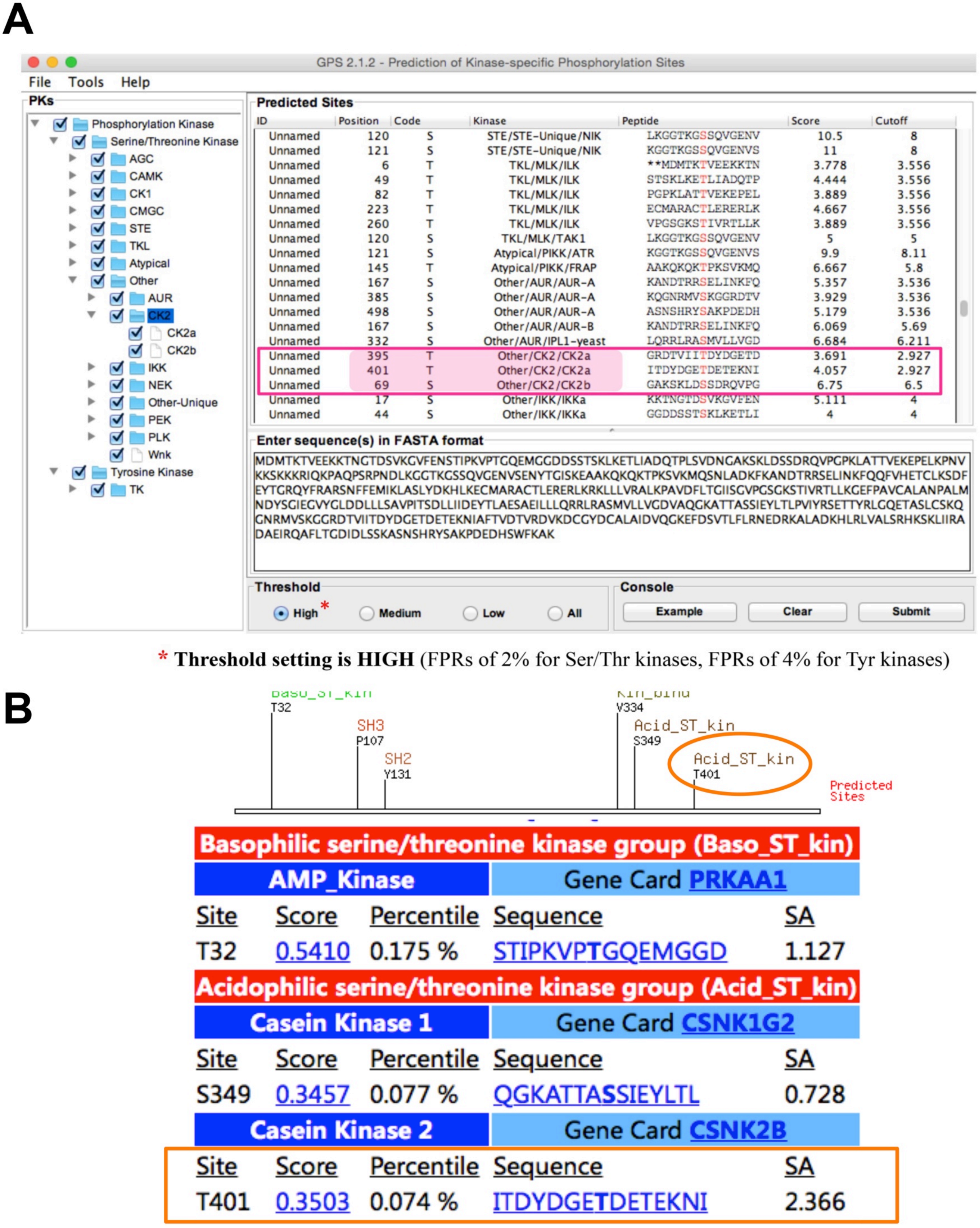

Fig. S1 Phosphorylation predictions of the $_{XJ}$TGB1 protein by the GPS 2.1 program (A) and the Scansite Motif Scanner online server (B).

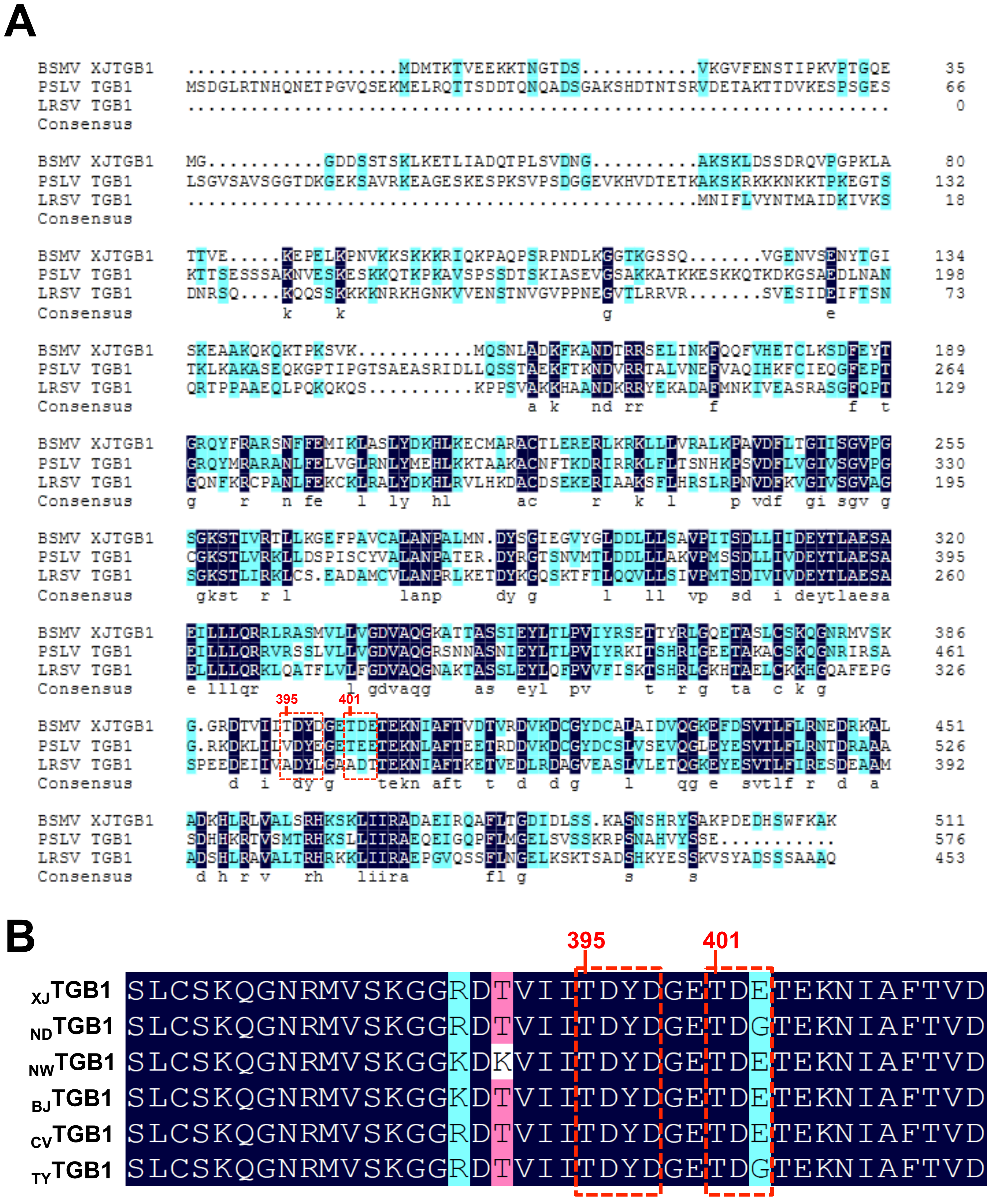

Fig. S2 Alignment of the TGB1 proteins of the hordeiviruses (A) and amongst six sequenced BSMV strains (B).

Table S1 Primers used in construction and analysis of biologically active BSMV Xinjiang cDNA clones

Primer	Primer sequence (5'-3') [a]	Position and description [b]	Purpose
XJ-1	TGTAATACGACTCACTATAGGTATGTAAGTTGCCTTTGGGTG	${}_{XJ}$BSMV RNAα nt 1-22	pT7α_{XJ} construction in combination with BS-32
XJ-2	TGTAATACGACTCACTATAGGTAAAAGAAAAGGAACAACCCTG	${}_{XJ}$BSMV RNAβ nt 1-23	pT7β_{XJ} construction in combination with BS-32
XJ-3	TGTAATACGACTCACTATAGGTATAGCTTGAGCATTACCGTC	${}_{XJ}$BSMV RNAγ nt 1-22	pT7γ_{XJ} construction in combination with BS-32
CH-10	GTATGTAAGTTGCCTTTGGGTGTA	${}_{XJ}$BSMV RNAα nt 1-24	pCaBS-α_{XJ} construction in combination with BS-26
CH-11	GTAAAAGAAAAGGAACAACCCTGT	${}_{XJ}$BSMV RNAβ nt 1-24	pCaBS-β_{XJ} and its mutants construction in combination with BS-26
CH-12	GTATAGCTTGAGCATTACCGTCGT	${}_{XJ}$BSMV RNAγ nt 1-24	pCaBS-γ_{XJ} construction in combination with BS-26
BS-26	CGGGATCCTGGTCTTCCCTTGGGGGAC	${}_{XJ}$BSMV RNAγ nt 2793-2775 (common to the 3' termini of BSMV gRNAs)	pCaBS-α_{XJ}, pCaBS-β_{XJ}, and pCaBS-γ_{XJ} construction
BS-10	GGTGCTTGATGCTTTGGATAAGG	${}_{XJ}$BSMV RNAγ nt 1864-1886	verification of BSMV genome by RT-PCR
BS-32	TGGTCTTCCCTTGGGGGAC	${}_{XJ}$BSMV RNAγ nt 2790-2772	
TGB1-XhoIF	CCGCTCGAGCTATGGACATGACGAAAACTGTTGAGG	${}_{XJ}$TGB1 nt 1-25	pGDG-${}_{XJ}$TGB1and its mutants construction
TGB1-BamHIR	CGGGATCCTTATTTGGCCTTGAACCAACTGTGG	${}_{XJ}$TGB1 nt 1536-1512	
TGB2-XhoIF	CCGCTCGAGCTATGAAGACCACAGTTG	${}_{XJ}$TGB2 nt 1-16	pGD-${}_{XJ}$TGB2 construction
TGB2-BamHIR	CGGGATCCCTAGCCAATATCGCATAG	${}_{XJ}$TGB2 nt 395-379	

Primer	Primer sequence (5'-3') [a]	Position and description [b]	Purpose
TGB3-XhoIF	CCGCTCGAGCTATGGCAATGCCTCATC	$_{XJ}$TGB3 nt 1-16	pGDG-$_{XJ}$TGB3 and pGDR-$_{XJ}$TGB3 construction
TGB3-BamHIR	CGGGATCCTTACCTTTTTGAAGAAAG	$_{XJ}$TGB3 nt 469-451	
TGB1-NdeIF	GGAATTCCATATGATGGACATGACGAAAACTG	$_{XJ}$TGB1 nt 1-19	pET-$_{XJ}$TGB1 construction
TGB1-XhoIR	CCGCTCGAGTTTGGCCTTGAACCAAC	$_{XJ}$TGB1 nt 1533-1517	
TGB3-BamHIF	CGGGATCCATGGCAATGCCTCATC	$_{XJ}$TGB3 nt 1-16	pGEX-$_{XJ}$TGB3 construction
TGB3-XhoIR	CCGCTCGAGTTACCTTTTTGAAGAAAG	$_{XJ}$TGB3 nt 468-451	
NbCK2α-NdeIF	GGAATTCCATATGATGTCAAAAGCTCGTG	NbCK2α nt 1-16	pET- NbCK2α construction
NbCK2α-XhoIR	CAACTCGAGCTGCGTCCTCATCC	NbCK2α nt 999-986	
HvCK2α-NdeIF	GGAATTCCATATGATGTCGAAGGCGAGGGTC	HvCK2α nt 148-165	pET- HvCK2α construction
HvCK2α-SalIR	GCGTCGACTTGCGGTCGTGC	HvCK2α nt 1146-1135	

[a] Underlined letters indicate restriction enzyme sites, gray covered letters show T7 promoter sequence.

[b] Numbers correspond to target nucleotide positions; a reverse order of numbers indicates that the primer is complementary to the targeted sequences.

Table S2 Primers used for site-specific mutagenesis of Xinjiang RNAβ clones

Primer	Primer sequence (5'-3') [a]	Position and description [b]	Purpose
T395A-F	GGGACACAGTGATCATT**G**CTGATTACGATGGCG	$_{XJ}$BSMV RNAβ nt 1958-1990	pT7β$_{XJ}$TGB1$_{T395A}$ and pET-$_{XJ}$TGB1$_{T395A}$ construction
T395A-R	CGCCATCGTAATCAG**C**AATGATCACTGTGTCCC	$_{XJ}$BSMV RNAβ nt 1990-1958	
T395D-F	GGAAGGGACACAGTGATCATT**GA**TGATTACGATGGCGAAACAG	$_{XJ}$BSMV RNAβ nt 1954-1996	pT7β$_{XJ}$TGB1$_{T395D}$ and pET-$_{XJ}$TGB1$_{T395D}$ construction
T395D-R	CTGTTTCGCCATCGTAATCA**TC**AATGATCACTGTGTCCCTTCC	$_{XJ}$BSMV RNAβ nt 1996-1954	
T395E-F	GGAAGGGACACAGTGATCATT**GAA**GATTACGATGGCGAAACAG	$_{XJ}$BSMV RNAβ nt 1954-1996	pT7β$_{XJ}$TGB1$_{T395E}$ and pET-$_{XJ}$TGB1$_{T395E}$ construction
T395E-R	CTGTTTCGCCATCGTAATC**TTC**AATGATCACTGTGTCCCTTCC	$_{XJ}$BSMV RNAβ nt 1996-1954	
T401A-F	GATTACGATGGCGAA**G**CAGATGAAACGGAGAAAAATATCG	$_{XJ}$BSMV RNAβ nt 1978-2017	pT7β$_{XJ}$TGB1$_{T401A}$ and pET-$_{XJ}$TGB1$_{T401A}$ construction
T401A-R	CGATATTTTTCTCCGTTTCATCTG**C**TTCGCCATCGTAATC	$_{XJ}$BSMV RNAβ nt 2017-1978	
T401D-F	GATTACGATGGCGAA**GAC**GATGAAACGGAGAAAAATATCG	$_{XJ}$BSMV RNAβ nt 1978-2017	pT7β$_{XJ}$TGB1$_{T401D}$ and pET-$_{XJ}$TGB1$_{T401D}$ construction
T401D-R	CGATATTTTTCTCCGTTTCATC**GTC**TTCGCCATCGTAATC	$_{XJ}$BSMV RNAβ nt 2017-1978	
T401E-F	GATTACGATGGCGAA**GA**AGATGAAACGGAGAAAAATATCG	$_{XJ}$BSMV RNAβ nt 1978-2017	pT7β$_{XJ}$TGB1$_{T401E}$ and pET-$_{XJ}$TGB1$_{T401E}$ construction
T401E-R	CGATATTTTTCTCCGTTTCAT**CT**TCTTCGCCATCGTAATC	$_{XJ}$BSMV RNAβ nt 2017-1978	
T395A/ T401A -F	CACAGTGATCATT**G**CTGATTACGATGGCGAA**G**CAGATGAAACGG	$_{XJ}$BSMV RNAβ nt 1962-2005	pT7β$_{XJ}$TGB1$_{T395A/T401A}$ and pET-$_{XJ}$TGB1$_{T395A/T401A}$ construction
T395A/ T401A -R	CCGTTTCATCTG**C**TTCGCCATCGTAATCAG**C**AATGATCACTGTG	$_{XJ}$BSMV RNAβ nt 2005-1962	
T395D/ T401A -F	CACAGTGATCATT**GA**TGATTACGATGGCGAA**G**CAGATGAAACGG	$_{XJ}$BSMV RNAβ nt 1962-2005	pT7β$_{XJ}$TGB1$_{T395D/T401A}$ and pET-$_{XJ}$TGB1$_{T395D/T401A}$ construction
T395D/ T401A -R	CCGTTTCATCTG**C**TTCGCCATCGTAATCA**TC**AATGATCACTGTG	$_{XJ}$BSMV RNAβ nt 2005-1962	

T395E/ T401A -F	CACAGTGATCATT**GAA**GATTACGATGGCGAA**G**CAGATGAAACGG	$_{XJ}$BSMV RNAβ nt 1962-2005	pT7β$_{XJ}$TGB1$_{T395E/T401A}$ and pET-$_{XJ}$TGB1$_{T395E/T401A}$ construction
T395E/ T401A -R	CCGTTTCATCTG**C**TTCGCCATCGTAATC**TTC**AATGATCACTGTG	$_{XJ}$BSMV RNAβ nt 2005-1962	

[a] Underlined and bold letters indicate sequences altered by site-specific mutagenesis.

[b] Numbers correspond to target nucleotide positions; a reverse order of numbers indicates that the primer is complementary to the targeted sequences.

Table S3 Sequence alignment of Xinjiang strain RNAα with different BSMV strains

**Identity of sequences (%)**			
Strain	Full length	αa	
	nt	nt	aa
ND18	99.82	99.85	99.65
Type	99.66	99.82	99.56
Norwich	98.52	98.51	98.77
Beijing	95.18	94.96	96.66

Table S4 Sequence alignment of Xinjiang strain RNAβ with different BSMV strains

Identity of sequences (%)								
Strain	Full length	βa		TGB1		TGB2		TGB3	
	nt	nt	aa	nt	aa	nt	aa	nt	aa
ND18	99.23	99.83	99.49	99.67	99.41	100	100	99.78	100
Type	99.41	100	100	99.67	99.22	99.75	100	100	100
Norwich	95.85	99.16	100	95.72	93	97.2	99.24	95.27	93.55
Beijing	94.08	92.42	92.42	95.39	93.76	96.69	99.24	96.13	94.19

Table S5 Sequence alignment of Xinjiang strain RNAγ with different BSMV strains

Identity of sequences (%)					
Strain	Full length	γa		γb	
	nt	nt	aa	nt	aa
ND18	98.78	98.92	99.38	97.6	96.73
Type	87.82	83.74	83.83	99.35	99.35
Norwich	98.5	98.77	99.54	97.42	98.69
Beijing	94.36	95.02	96.92	94.55	94.12

Table S6 Systemic infectivity efficiency of $_{XJ}$BSMV phosphorylation related mutants on hosts

	Mock	WT	T395A	T395D	T395E	T401A	T401D	T401E	T395A T401A	T395D T401A	T395E T401A
**Systemic infectivity efficiency on *Nicotiana benthamiana***											
Exp. 1	0[a]/8[b]	7/8	6/8	0/8	0/8	0/8	0/8	0/8	0/8	0/8	0/8
Exp. 2	0/8	7/8	6/8	0/8	0/8	0/8	0/8	0/8	0/8	0/8	0/8
Exp. 3	0/8	7/8	5/8	0/8	0/8	0/8	0/8	0/8	0/8	0/8	0/8
**Systemic infectivity efficiency on barley**											
Exp. 1	0/8	6/8	5/8	0/8	4/8	1/8	0/8	4/8	0/8	0/8	0/8
Exp. 2	0/8	6/8	3/8	0/8	2/8	2/8	0/8	1/8	0/8	0/8	0/8
Exp. 3	0/8	6/8	4/8	0/8	4/8	1/8	0/8	4/8	0/8	0/8	0/8
**Systemic infectivity efficiency on wheat**											
Exp. 1	0/8	7/8	6/8	0/8	6/8	4/8	0/8	5/8	0/8	0/8	0/8
Exp. 2	0/8	7/8	5/8	0/8	5/8	3/8	0/8	5/8	0/8	0/8	0/8
Exp. 3	0/8	8/8	6/8	0/8	5/8	2/8	0/8	4/8	0/8	0/8	0/8

[a] numbers of systemically infected plants
[b] numbers of the total inoculated plants
